# The Antibacterial Activity of *Cassia fistula* Organic Extracts

**DOI:** 10.5812/jjm.8921

**Published:** 2014-01-01

**Authors:** Seyyed Mansour Seyyednejad, Hossein Motamedi, Mouzhan Vafei, Ameneh Bakhtiari

**Affiliations:** 1 Department of Biology, Faculty of Science, Shahid Chamran University, Ahvaz, IR Iran

**Keywords:** *Cassia fistula*, Anti-bacterial Activity, Disc Diffusion Antibacterial Test, Minimum Inhibitory Concentration, Minimum Bactericidal Concentration

## Abstract

**Background:**

*Cassia fistula*, is a flowering plant and a member of *Fabaceae* family. Its leaves are compound of 4 - 8 pairs of opposite leaflets. There are many *Cassia* species around the world which are used in herbal medicine.

**Objectives:**

This study was designed to examine *in vitro* anti-bacterial activity of methanolic and ethanolic extracts of *C. fistula* native to Khuzestan, Iran.

**Materials and Methods:**

The microbial inhibitory effect of methanolic and ethanolic extracts of *C. fistula* was tested on 3 Gram positive: *Bacillus cereus, Staphylococcus aureus* and *S*.* epidermidis* and 5 Gram negative: *Salmonella Typhi*, *Kelebsiella pneumoniae*, *Escherichia coli*, *Pseudomonas aeruginosa*, and* Proteus mirabilis* bacterial species using disc diffusion method at various concentrations. The minimum inhibitory and bactericidal concentrations (MIC and MBC) were measured by the tube dilution assay.

**Results:**

The extract of *C. fistula* was effective against *B. cereus, S. aureus, S. epidermidis*, *E. coli* and *K. pneumoniae*. The most susceptible microorganisms to ethanolic and methanolic extracts were *E. coli *and *K. pneumoniae*, respectively. Also *B. cereus* and *S. aureus* showed the least sensitivity to ethanolic and methanolic extracts, respectively. The MIC (minimum inhibitory concentration) and MBC (minimum bactericidal concentration) of ethanolic extracts against *S. aureus*,* E. coli*, *S. epidermidis *and *K. pneumoniae* were also determined.

**Conclusions:**

With respect to the obtained results and regarding to the daily increase of the resistant microbial strains to the commercial antibiotics, it can be concluded that these extracts can be proper candidates of antibacterial substance against pathogenic bacterial species especially *S. aureus, E. coli*, *K. pneumoniae* and *S. epidermidis*.

## 1. Background

Since the introduction of the antibiotics, they have been known as one of the most important tools against bacterial infections (1). Although, in the past years due to the widespread and in some cases incorrect use of antibiotics we can see dramatic increase in microbial resistance against antimicrobial agents that is a critical reason for finding new drugs with les resistance and side effects (1). The medicinal plants have been used from ancient times especially in Asia, and have been used for the treatment of specific illness (2). In Iran, we can find the history of using these plants in Ibn-Sina (Avicenna, 980-1037) books (3, 4). They contain active components used in the treatment of many human diseases. Many studies showed that some plants contain many components like peptides, unsaturated long chain aldehydes, alkaloid constituents, some essential oils, phenols and soluble compounds in water, ethanol, chloroform, methanol and butanol (5-7). 

*Cassia fistula *(*Leguminosae*), is distributed in different parts of the world including Asia, South Africa, Mexico, China, East Africa and Brazil. It is an ornamental tree with beautiful bunches of yellow flowers (8). It has been explained in the Indian literatures that *C. fistula *has advantageous application against some diseases such as skin infection, liver troubles and tuberculous glands and even this plant is used as a drug for rheumatism treatment, hematemesis, pruritus, leucoderma, and diabetes. Also Considerable effects of *C. fistula *against some microbes have been observed. With respect to these properties, this plant is used as broad-spectrum antimicrobial agent for treatment of some infectious diseases (8, 9).

Bacterial infectious diseases are widespread in Khuzestan (southwest of Iran) meanwhile different studies have reported the emergence of antibiotic resistant strains from this province. Actually, the raise of antibiotic resistant pathogens is a growing concern all over the world and WHO assign it as an emerging public health problem (10). For example some bacteria such as *Escherichia coli* and *Klebsiella* spp., the main agents of UTI (urinary tract infection), had high resistance to cephalosporins that is resulted from the production of extended-spectrum beta-lactamases (ESBL). The EBSLs genes are located on transferable plasmids and consequently their distribution will be at high rate (11).

## 2. Objectives

The aim of this study was to discover the antimicrobial effects of *C. fistula *flowers, a native plant in Khuzestan, and comparing its properties with commonly used antibiotics. These results can be useful in finding new antibacterial agents.

## 3. Materials and Methods

### 3.1. Plant Collection and Identification

*C. fistula *was freshly collected in April 2011 from Shahid Chamran University farmlands, Ahvaz, Khuzestan province. The taxonomic identification of this plant performed by comparison with existing herbarium in Biology department of Shahid Chamran University.

### 3.2. Extract Preparation

The flowers of *C. fistula *were shade dried at room temperature for 7 days and then finely powdered using electronic blender. One gram of powder was extracted using 10 mL of alcohol (ethanol or methanol)-distilled water solution (8:2 v/v), centrifugation (3000 rpm) for 15 minutes and finally harvesting the supernatant (12, 13). This process was repeated for three times. Eventually the extracts were placed at room temperature to evaporate the solvent (12, 13).

### 3.3. Bacterial Test

A total of 8 bacterial species were tested including 3 Gram positive: *Bacillus cereus, Staphylococcus aureus *and* S*.* epidermidis *and 5 Gram negative: *Salmonella Typhi, Kelebsiella pneumoniae, E*.* coli, Pseudomonas aeruginosa, *and *Proteus mirabilis*. These species were originally isolated from the clinical samples and identified based on standard phenotypic tests according to Bergey’s manual of systematic bacteriology.

### 3.4. Determination of Antibacterial Activity

Antibacterial activity of the ethanolic and methanolic extracts of the plant were studied by standard paper disc-diffusion method (14). Stock cultures of the bacteria were grown in Muller Hinton broth (MHB, Merck, Germany) medium at 37^˚^C for 22 hours. Final cell concentrations were adjusted to 10^5^ CFU.mL^-1^ with reference to the McFarland turbidometry (15). A lawn culture was prepared from bacterial suspension on Muller-Hinton agar (MHA, Merck, Germany) using sterile cotton swab and allowed to remain in contact for 1 minute. Five concentrations of ethanolic and methanolic extracts were prepared as follows 0.05, 0.1, 0.2, 0.4 and 0.6 g/mL. Sterile 6 mm filter paper discs were saturated with 30 µL of different concentrations of extracts (16, 17). So the effective dose of each disc was reached to 1.5, 3, 6, 12 and 18 mg, respectively. Then it was allowed to evaporate the solvents and the discs were placed on bacterial cultures. The plates were left at room temperature for 1 hour, then the petri dishes were subsequently incubated at 37˚C for 24 hours and the inhibition zone of each disc was measured in millimeter.

### 3.5. Determination of Minimum Inhibitory Concentrations 

The minimum inhibitory concentration (MIC) of the extracts was determined for most sensitive bacterial species. A 16-hour culture was diluted with a sterile physiologic saline solution (0.9% (w/v) sodium chloride) with reference to the 0.5 McFarland turbidometry to achievement the inoculum approximately equal to 10^5^ CFU.mL^-1^ (15). In the tube dilution assay, standard bacterial suspension and different concentration of extracts (5, 10, 20, 40, 80 and 160 mg/mL) were added to tubes containing 1 mL Muller Hinton broth. These tubes were incubated at 37°C for 24 hours. The first tube of the series with no sign of visible growth was considered as the MIC. This process has been done three times (18).

### 3.6. Determination of Minimum Bactericidal Concentrations 

To determine the MBC, for each set of test tubes in the MIC assay, a loop full of broth was collected from the tubes without any visible growth and cultured at 37°C for 18 - 24 hours. The highest dilution that yields no colony formation on solid medium was considered as MBC (19).

### 3.7. Time-Kill Kinetic Study

The time-kill kinetics was studied by culturing one standard loop of the suspension from the tube possessing MBC on MHA from 0 to 36 hours. This was performed at the first hours of intervals for the first 18-hour study, and then at 2-hour intervals for the later 18 hours (18). 

## 4. Results

Both alcoholic extracts of *C. fistula *exhibited antibacterial activity against Gram positive and Gram negative species. As a result, both alcoholic extracts efficiently inhibited three Gram positive species including *S. aureus*, *S. epidermidis* and *B. cereus* and two Gram negative bacteria including *E .coli *and *K. pneumoniae*. The antimicrobial activity results were shown in Tables 1 and 2. The highest activity (with inhibition zone diameter about 26 mm) was demonstrated in case of ethanolic extract of *C. fistula *flowers against *E. coli* while the lowest activity (with inhibition zone diameter about 7 mm) was demonstrated by the ethanolic extract against *B. cereus* and* S. aureus* and methanolic extract against *B. cereus *and *S. epidermidis*. On the other hand, the ethanolic and methanolic extracts were not active against *S. *Typhi*, P. aeruginosa, K. pneumoniae* and *P. mirabilis.* The results of MIC and MBC of ethanolic and methanolic extracts for 3 bacterial species were shown in Table 3. Time-kill kinetic of ethanolic extract of *C. fistula *was 4 hours (Table 4). 

**Table 1.** Results of Antibacterial Activity of Ethanolic and Methanolic Extracts of *Cassia fistula* Flowers

	Ethanolic Extract (Effective dose, mg)					Methanolic Extract (Effective Dose, mg)				
**Bacterial species**	18	12	6	3	1.5	18	12	6	3	1.5
**Gram positive**										
*B. cereus*	9^a^	8	7	-	-	8	7	-	-	-
*S. aureus*	13	10	9	7	-	12	10	8	-	-
*S. epidermidis*	15	14	11	9	-	13	13	9	7	-
**Gram negative**										
*E. coli*	26	20	17	13	11	22	20	18	14	10
*K. pneumoniae*	23	22	19	16	12	25	22	18	17	13

^a^ Diameter of Inhibition zone (mm), disc diameter: 6.4 mm.

**Table 2.** Inhibition Zone^a^ of Standard Antibiotics on Tested Bacteria

Antibiotic Disc									
Bacterial species	NF^b^	CB^b^	NB^b^	DX^b^	OX^b^	Van^b^	Cef^b^	Tet^b^	Pen^b^
**Gram positive**									
*S. aureus*	R	13	31	15	R	15	25	20	30
*B. cereus*	R	7	18	18	R	15	12	14	R
*S. epidermidis*	R	36	29	21	R	_	_	_	_
**Gram negative**									
*E. coli*	R	R	17	11	R	_	_	_	_
*K. pneumoniae*	R	R	11	R	_	R	13	R	R
*P. aeruginosa*	R	R	16	R	R	R	R	R	R
*S. Typhi*	R	27	34	30	R	_	_	_	_
*P .mirabilis*	_	_	_	_	_	R	19	R	15

^a^ Diameter of Disc: 6 mm

^b^ Abbreviations: NF, Nafcillin 1 mcg; CB, Carbenicillin 100 mcg; NB, Novobiocin 30 mcg; DX, Doxycycline 30 mcg; CL, Colistin 10 mcg; MT, Methicillin 5 mcg; OX, Oxacycline 1 mcg; R, Resistance

**Table 3.** Antibacterial Activity (MIC and MBC, mg.mL ^-1^) of the Ethanolic and Methanolic Extracts of *Cassia fistula*

	Ethanolic				Methanolic		
	*S. aureus*	*E. coli*	*S. epidermidis*	*K.pneumoniae*	*S. aureus*	*E.coli*	*K.pneumoniae*
**MIC**	40	5	40	5	20	10	5
**MBC**	….	40	….	160	40	20	160

**Table 4.** The Time-Kill Kinetic of Methanolic Extract of *C*.* fistula* at 20 mg/mL Concentration Against *E. coil*

Hour	1	2	3	4	5	6	7	8	9	10	11	12	13	14	15	16	17	18	20	22	24	26	28	30	32	34	36
**Ethanolic Extract**	+^a^	+	+	-^b^	-	-	-	-	-	-	-	-	-	-	-	-	-	-	-	-	-	-	-	-	-	-	**-**

^a^ (+): Growth

^b^ (-): Growth Inhibition

## 5. Discussion

About 80% of world population use plant extracts or their active components in traditional therapies (1). In this study, we have studied the ethanolic and methanolic extracts obtained from *C. fistula *flowers. Both extracts showed significant activity against five of the tested strains. Interestingly, among sensitive strains, the Gram negative bacteria were more sensitive than Gram positive ones. From this finding it can be concluded that the possible target site of these extracts is the structures except the cell wall. It may be affect the outer membrane of Gram negative bacteria or protein synthesis mechanisms. 

In any case, these extract can be a proper candidate using against infections caused by Gram negative bacteria. Furthermore, there was a direct relationship between effective dose of these extracts and their antibacterial effects. Also, in some cases, they showed an effective dose 1.5 mg which can be a significant finding. These antibacterial activities were comparable to routinely used antibiotics. Most of the antimicrobial effects of *C. fistula *is related to their components and secondary metabolites like phenolic compounds (20, 21). Phytochemical studies showed that this plant containing components like saponin, triterpnoids, glycosides, anthraquinore, steroids and flavonoids that inhibit the growth of the tested bacterial strains (22, 23). The presence of this component inhibits the growth of tested bacterial strains. 

The antimicrobial activity depends on the contents of phenolic components of the plant extracts. High amounts of phenolic group in the aerial parts of *C. fistula *implied that these components may be the active compounds, which may be responsible for the antibacterial activity (24). Rizvi et al. observed that *Cassia* species had a significant activity against Gram positive microorganisms. They claimed that this is the result of some substances like flavonoids and polysaccharides (23). Abo et al. also found that leaves extracts of *C. fistula* have considerable antimicrobial activity (25). Vasudevan et al. reported that methanolic extracts inhibited Gram positive bacteria more than Gram negative species (24). The result of this study suggests that *C. fistula *extracts can be useful to treat infectious diseases and must be considered as a new source of antibacterial agents.

## References

[1] Bhalodia NR, Shukla VJ (2011). Antibacterial and antifungal activities from leaf extracts of Cassia fistula l.: An ethnomedicinal plant.. J Adv Pharm Technol Res..

[2] Bhattacharjee Supriya Kumar (2000). Handbook of medicinal plants..

[3] Lotfipour F, NAZEMIEH H, FATHIAZAD F, Garaei N, Arami S, Talat S (2008.). Evaluation of antibacterial activities of some medicinal plants from North-West Iran.. Iran J Basic Med Sci..

[4] Motamedi H, Darabpour E, Gholipour M, Nejad SMSeyyed (2010). Antibacterial Effect of Ethanolic and Methanolic Extracts of Plantago ovata and Oliveria decumbens Endemic in Iran Against Some Pathogenic Bacteria.. Int J Pharmacol..

[5] Alma MH, Mavi A, Yildirim A, Digrak M, Hirata T (2003). Screening chemical composition and in vitro antioxidant and antimicrobial activities of the essential oils from Origanum syriacum L. growing in Turkey.. Biol Pharm Bull..

[6] Klausmeyer P, Chmurny GN, McCloud TG, Tucker KD, Shoemaker RH (2004). A novel antimicrobial indolizinium alkaloid from Aniba panurensis.. J Nat Prod..

[7] Seyyednejad SM, Maleki S, Damab NMirzaei, Motamedi H (2008). Antibacterial Activity of Prunus mahaleb and Parsley (Petroselinum crispum) Against Some Pathogen.. Asian J Biological Sci..

[8] Duraipandiyan V, Ignacimuthu S (2007). Antibacterial and antifungal activity of Cassia fistula L.: an ethnomedicinal plant.. J Ethnopharmacol..

[9] Panda SK, Padhi LP, Mohanty G (2011). Antibacterial activities and phytochemical analysis of Cassia fistula (Linn.) leaf.. J Adv Pharm Technol Res..

[10] Dallal Mohammad Mehdi Soltan, Taremi Mahnaz, Gachkar Latif, Modarressi Shabnam, Sanaei Maryam, Bakhtiari Rounak (2007). Characterization of antibiotic resistant patterns of Salmonella serotypes isolated from beef and chicken samples in Tehran.. Jundishapur J Microbiol..

[11] Irajian Golamreza, Jazayeri Moghadas Ali (2011). Frequency of extended-spectrum beta lactamase positive and multidrug resistance pattern in Gram-negative urinary isolates, Semnan, Iran.. Jundishapur J Microbiol..

[12] Moazedi AA, Mirzaie DN, Seyyednejad SM, Zadkarami MR, Amirzargar A (2007). Spasmolytic effect of Petroselinum crispum (Parsley) on rat's ileum at different calcium chloride concentrations.. Pak J Biol Sci..

[13] Seyyednejad M, Ebrahimzadeh H, Talaei A (2001). Carbohydrate content in olive Zard cv and alternate bearing pattern.. Int Sugar J..

[14] Seyyednejad SM, Koochak H, Darabpour E, Motamedi H (20101). A survey on Hibiscus rosa -sinensis, Alcea rosea L. and Malva neglecta Wallr as antibacterial agents.. Asian Pac J Trop Med..

[15] Burt SA, Reinders RD (2003). Antibacterial activity of selected plant essential oils against Escherichia coli O157:H7.. Lett Appl Microbiol..

[16] Cermelli C, Fabio A, Fabio G, Quaglio P (2008). Effect of eucalyptus essential oil on respiratory bacteria and viruses.. Curr Microbiol..

[17] Hsieh Pao-Chuan, Mau Jeng-Leun, Huang Shu-Hui (2001). Antimicrobial effect of various combinations of plant extracts.. Food Microbiol..

[18] Mahboobi Mohaddeseh, Shahcheraghi Fereshteh, Feizabadi Mohammad Mehdi (2006). Bactericidal effects of essential oiIs from clove, lavender and geranium on multi-drug resistant isolates of Pseudomonas aeruginosa.. Iran J Biotechnol..

[19] Motamedi H, Safary A, Maleki S, Seyyednejad SM (2009). Ziziphus spina-christi, a native plant from Khuzestan, Iran, as a potential source for discovery new antimicrobial agents.. Asian J Plant Sci..

[20] Aneja K, Sharma C, Joshi R (2011). In vitro efficacy of amaltas (Cassia fistula L.) against the pathogens causing otitis externa.. Jundishapur J Microbiol..

[21] Bahorun Theeshan, Neergheen Vidushi S, Aruoma Okezie I (2004). Phytochemical constituents of Cassia fistula.. Afr J Food Agr Nutr Develop..

[22] Draughon F Ann (2004). Use of botanicals as biopreservatives in foods.. Food Technol..

[23] Rizvi M Moshahid A, Irshad M, Hassadi GE, Younis Salaem Ben (2009). Bioefficacies of Cassia fistula: an Indian labrum.. Afr J Pharm Pharmacol..

[24] Vasudevan DEEPA T, Dinesh KAVITHA R, Gopalakrishnan S, Sreekanth SK, Shekar SONAL (2009). The potential of aqueous and isolated fraction from leaves of Cassia fistula Linn as antibacterial agent.. Int J Chem Sci..

[25] Abo KA, Lasaki SW, Adeyemi AA (1999). Laxative and antimicrobial properties of Cassia species growing in Ibadan.. Niger J Nat Prod Med..

